# Supplementary material for: Changes in Mitochondrial Size and Morphology in the RPE and Photoreceptors of the Developing and Ageing Zebrafish
Source: Cells. 2022 Nov 9;11(22):3542. doi: 10.3390/cells11223542 (PMC9688747; doi:10.3390/cells11223542)
Supplement: Supplementary file 1 [file cells-11-03542-s001.zip › cells-1886835-supplementary.pdf]

Supplementary data

Fission of megamitochondria into multiple smaller well-defined mitochondria in the ageing zebrafish retina

Thomas Burgoyne, Maria Toms, Chris Way, Dhani Tracey-White, Clare E. Futter, Mariya Moosajee

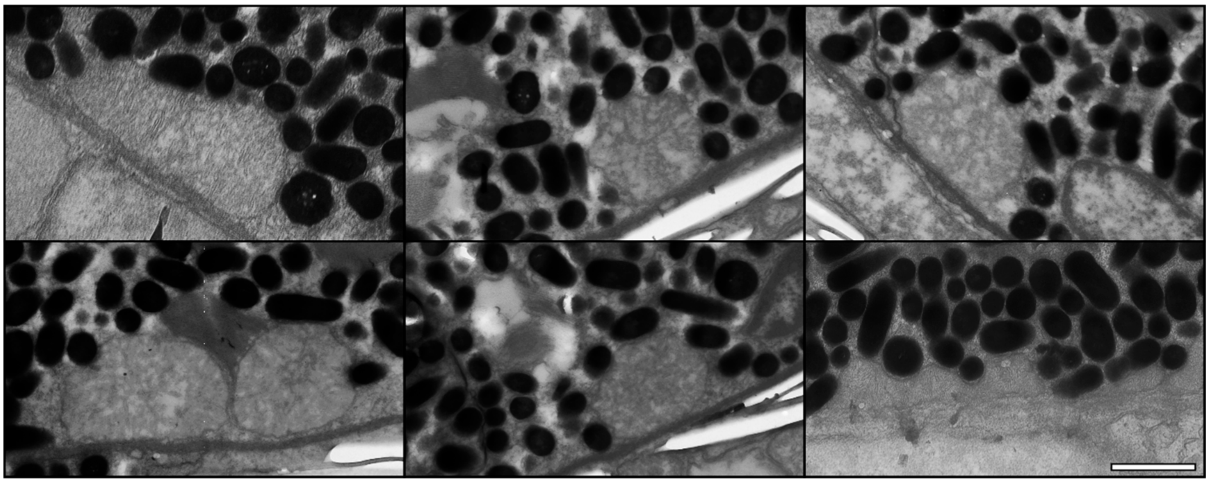

Figure S1. Further examples of mitochondria within the RPE at 5 days post-fertilisation (dpf). Scale bar = 1 μm.

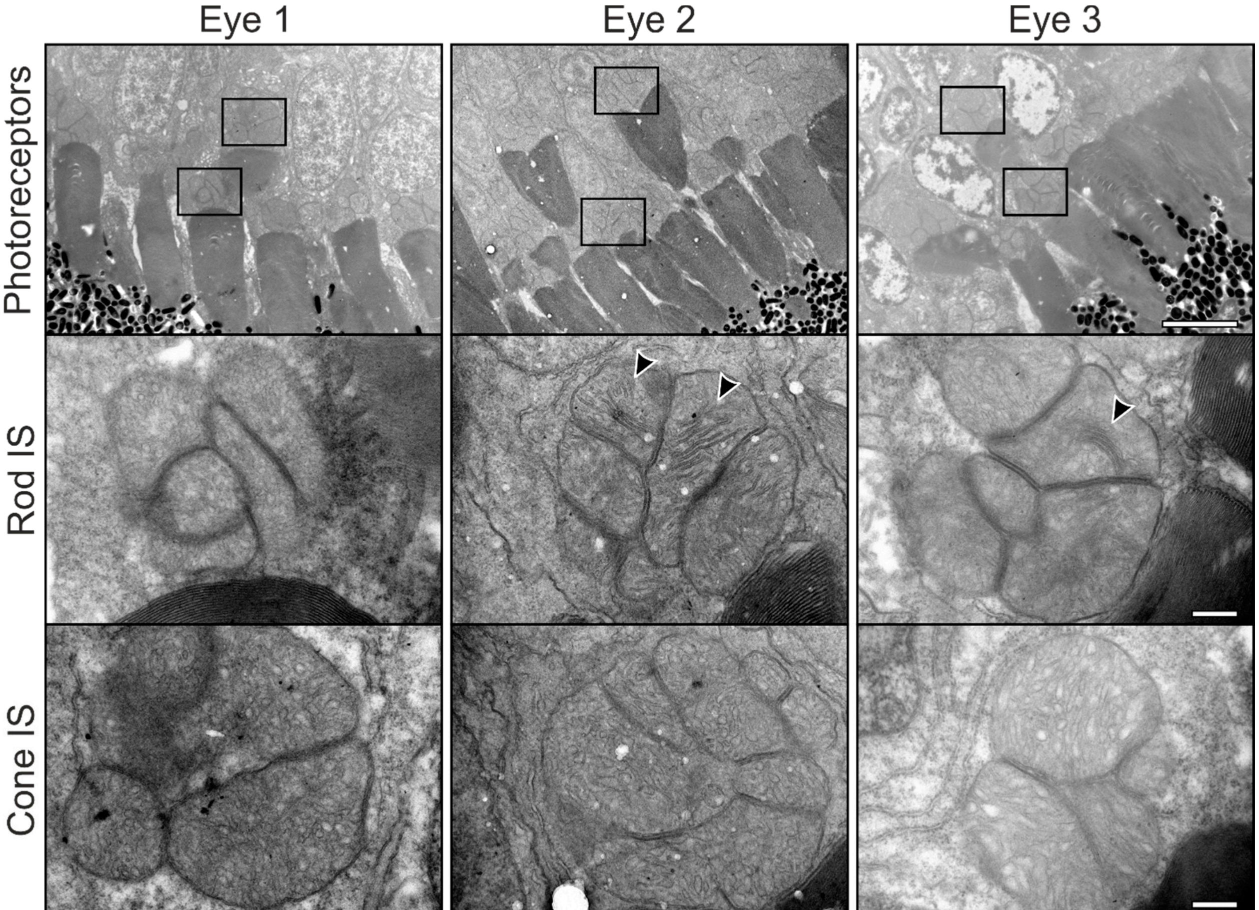

Figure S2. Electron microscopy images of mitochondria within the inner segment of rod and cone photoreceptors at 5 days post-fertilisation (dpf). These images were taken from eye acquired from three separate zebrafish. The black arrowheads indicate regions that have sheet like cristate within rod photoreceptors. Scale: Top row of panel 20 μm, and mid and bottom panels 200nm.

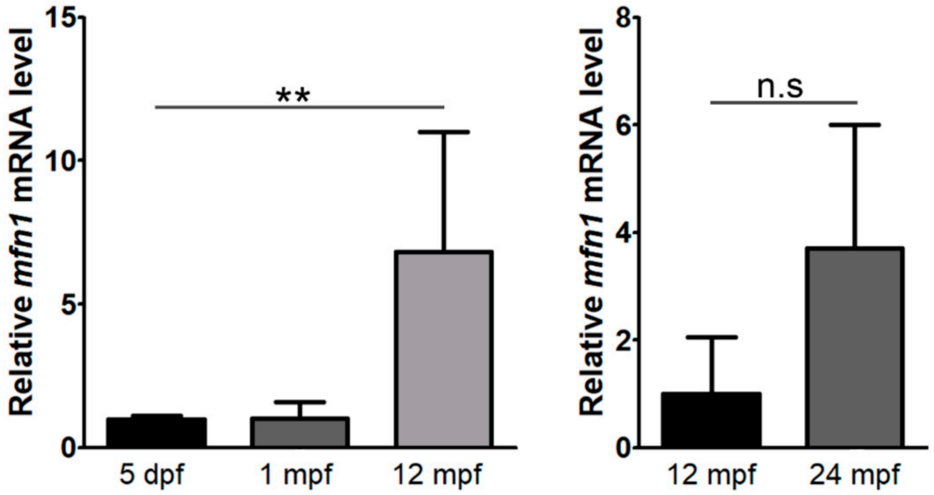

Figure S3. Expression levels of *mfn1* with age in the zebrafish retina. RT-qPCR was performed to assess expression of *mfn1* at 5 days post-fertilisation (dpf), 1 month post-fertilisation (mpf), 12 mpf and 24 mpf (n = 5). **p < 0.01; n.s, not significant.

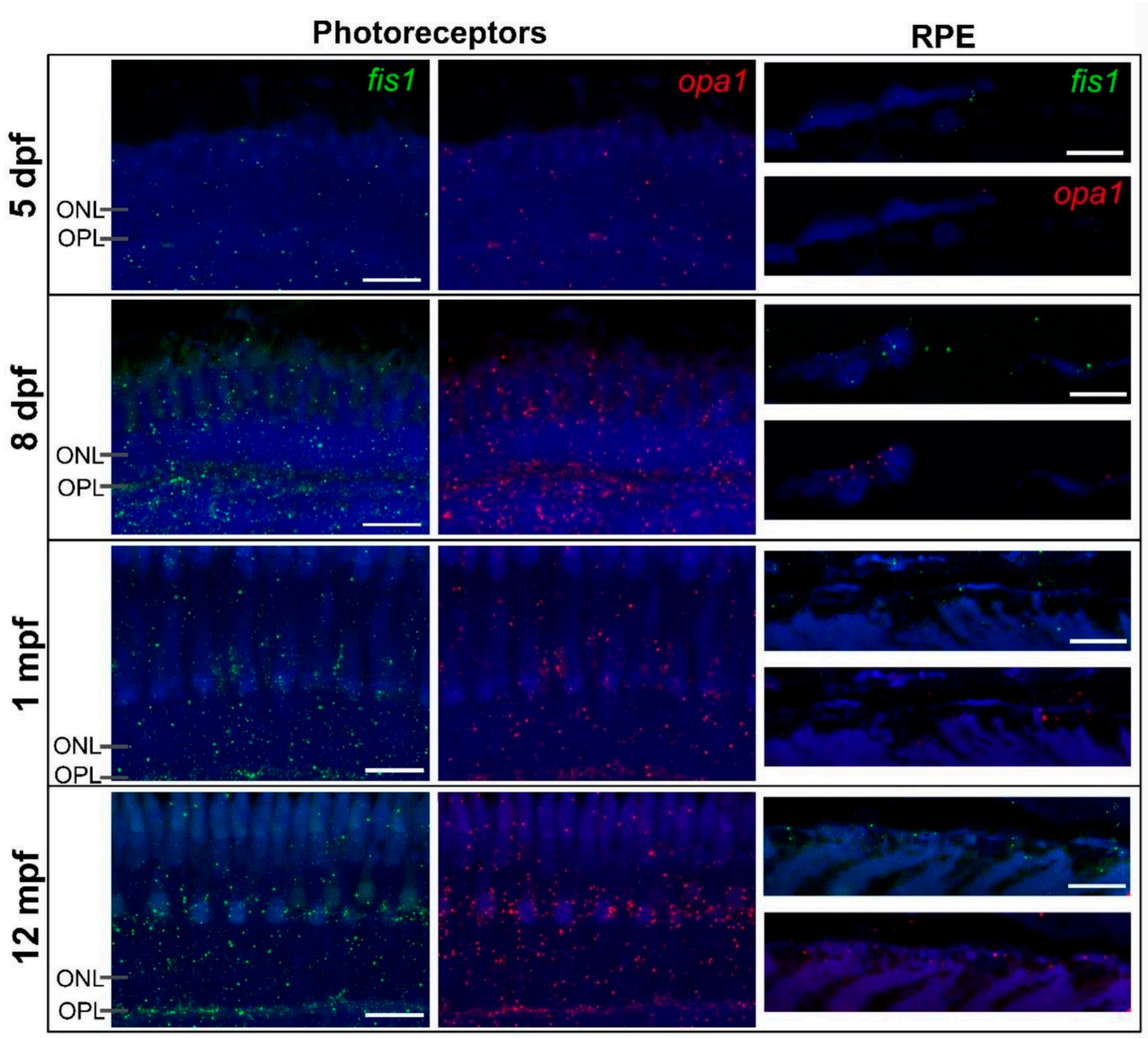

Figure S4. Expression of *fis1* (green) and *opa1*(red) mRNA in zebrafish photoreceptors and retinal pigment epithelium (RPE) at 5 days post-fertilisation (dpf), 8 dpf, 1 month post-fertilisation (mpf), and 12 mpf. mRNA transcripts were detected using the RNAscope fluorescent multiplex assay. ONL, outer nuclear layer; OPL, outer plexiform layer. Scale bar = 10 μm.
